# Supplementary material for: A comparative genomics methodology reveals a widespread family of membrane-disrupting T6SS effectors
Source: Nat Commun. 2020 Feb 27;11:1085. doi: 10.1038/s41467-020-14951-4 (PMC7046647; doi:10.1038/s41467-020-14951-4)
Supplement: Supplementary file 1 — Supplementary Information [file 41467_2020_14951_MOESM1_ESM.pdf]

Supplementary Information

A comparative genomics methodology reveals a widespread family of membrane-disrupting T6SS effectors

Chaya M. Fridman[1], Kinga Keppel[1], Motti Gerlic[1], Eran Bosis[2,*], and Dor Salomon[1,*]

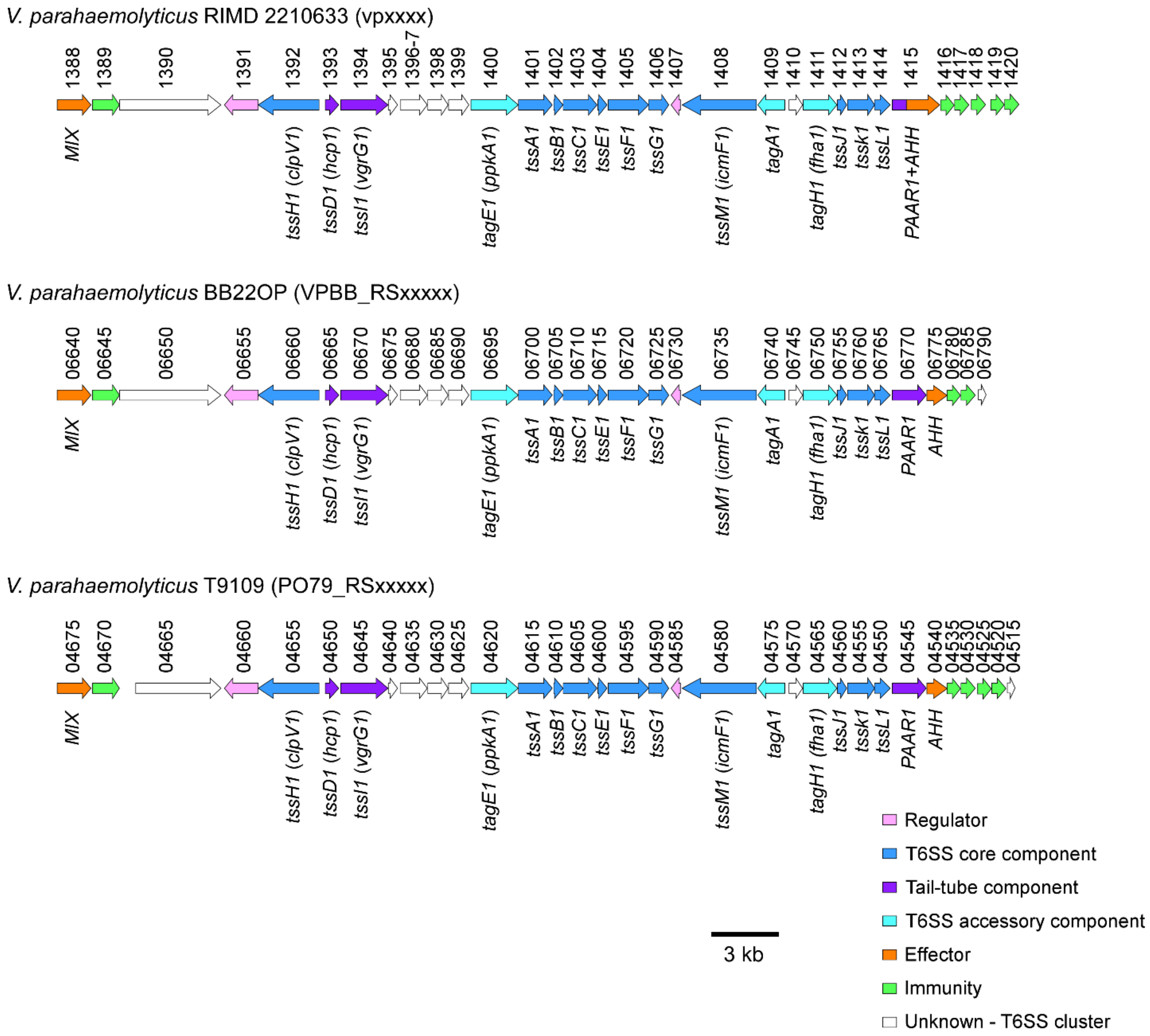

Supplementary Figure 1. The *V. parahaemolyticus* T6SS1 gene cluster. Gene structure of T6SS1 clusters in *V. parahaemolyticus* isolates RIMD 2210633, BB22OP, and T9019. Genes are represented by arrows indicating the direction of translation. Locus tags (vpxxx, VPBB_RSxxxx, and PO79_RSxxxx, respectively) are shown above, and gene names are shown below.

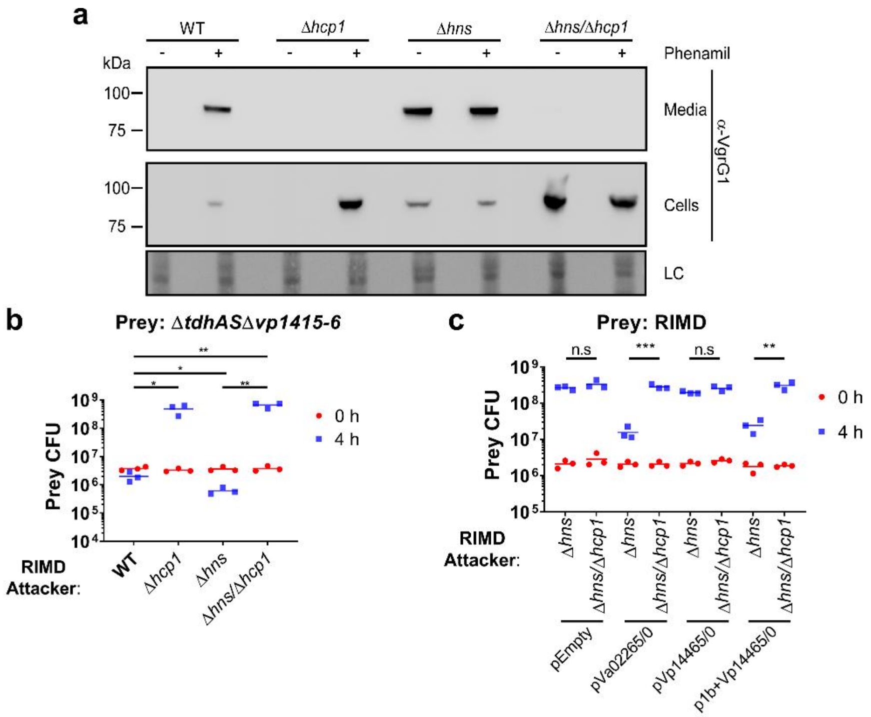

Supplementary Figure 2. The *V. parahaemolyticus* RIMD 2210633-based surrogate T6SS1 system. a, Expression (cells) and secretion (media) of VgrG1 from the indicated *V. parahaemolyticus* RIMD 2210633 mutants were detected by immunoblotting using specific antibodies against VgrG1. Loading control (LC), visualized as trihalo compounds' fluorescence of the immunoblot membrane, is shown for total protein lysates. Phenamil (20 µM) was added to induce surface sensing in suspension. The experiment was repeated three times with similar results. Results from a representative experiment are shown. **b,** Viability counts of *V. parahaemolyticus* RIMD 2210633 Δ*tdhAS*/Δ*vp1415-6* prey before (0 h) and after (4 h) co-incubation with the indicated *V. parahaemolyticus* RIMD 2210633 attackers at 30ºC (n = 3 co-cultures). Δ*vp1415-6* is a deletion of an E/I pair rendering the prey sensitive specifically to attackers delivering the T6SS1 VP1415 effector. Δ*hcp1* was used as a $T6SS^-$ control. **c,** Viability counts of *V. parahaemolyticus* RIMD 2210633 parental prey (RIMD) before (0 h) and after (4 h) co-incubation with surrogate system *V. parahaemolyticus* RIMD 2210633 attacker strains, Δ*hns* ($T6SS1^+$), and Δ*hns*/Δ*hcp1* ($T6SS1^-$) on media containing L-arabinose at 30ºC (n = 3 co-cultures). Attackers harbor plasmids for arabinose-inducible expression of the E/I pairs V12G01_02265/0 (pVa02265/0), B5C30_14465/0 (pVp14465/0), or VgrG1b+B5C30_14465/0 (p1b+Vp14465/0), or an empty vector (pEmpty). Asterisks denote the statistical significance between samples at the 4 h timepoint by unpaired, two-tailed Student's *t*-test (* $P < 0.05$; ** $P < 0.005$; *** $P < 0.0005$); n.s., no significant difference ($P > 0.05$). Source data are provided as a source data file.

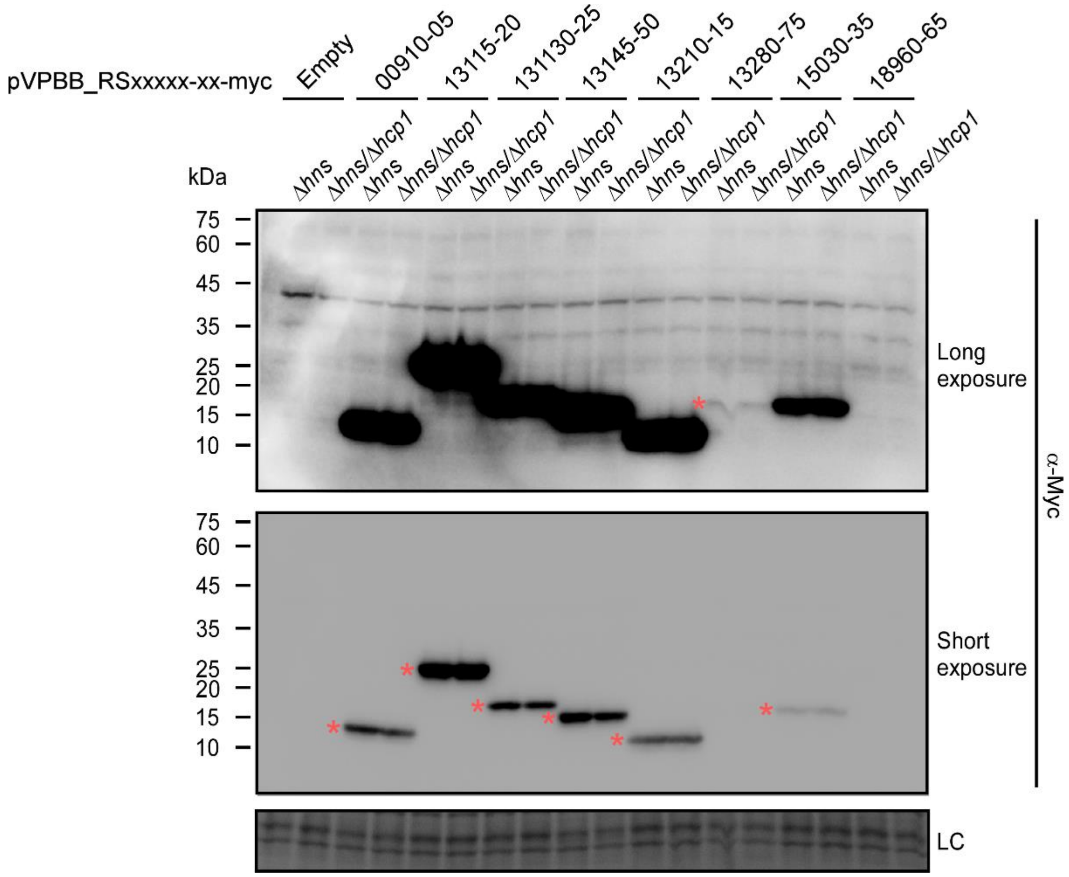

Supplementary Figure 3. The expression of candidate E/I cassettes from *V. parahaemolyticus* BB22OP. The expression of Myc-tagged, 3'-encoded protein of each *V. parahaemolyticus* BB22OP candidate effector and immunity pairs (as detailed in Supplementary Table 1) was detected by immunoblotting using anti-Myc antibodies. Indicated E/I pair were expressed in the surrogate system *V. parahaemolyticus* RIMD 2210633 strains Δ*hns* and Δ*hns*/Δ*hcp1* from an arabinose-inducible plasmid. The upper panel shows the longer exposure of a blot shown in the lower panel, to better visualize the low level expression of VPBB_RS13275. Red asterisks denote the bands of the expected size to their right. Loading control (LC), visualized as trihalo compounds' fluorescence of the immunoblot membrane, is shown for total protein lysates. Source data are provided as a source data file. The experiment was repeated three times with similar results. Results from a representative experiment are shown.

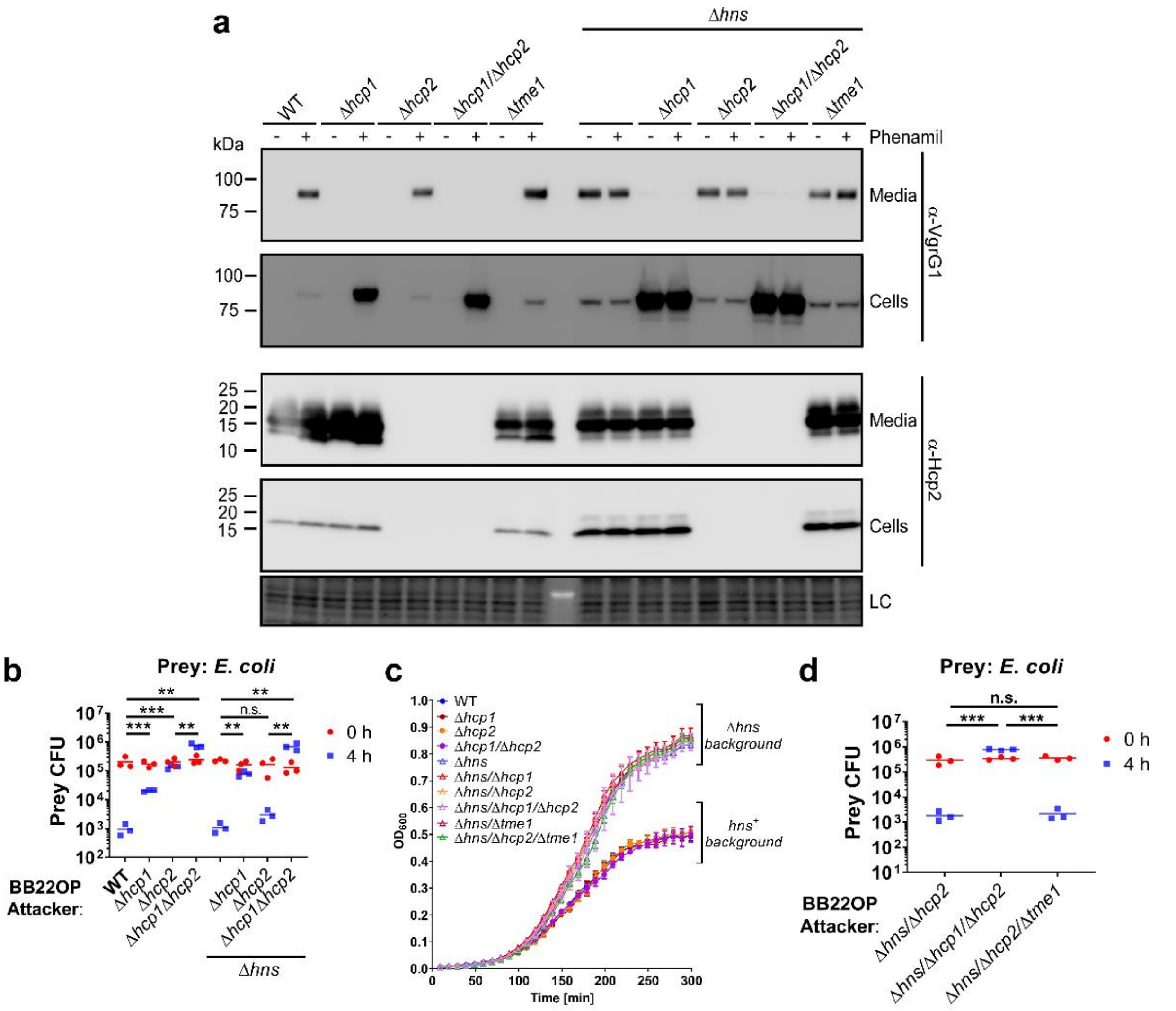

Supplementary Figure 4. BB22OP T6SS1 and T6SS2 are antibacterial systems that function under warm marine-like conditions. a, Expression (cells) and secretion (media) of VgrG1 and Hcp2 from the indicated *V. parahaemolyticus* BB22OP mutants were detected by immunoblotting using specific antibodies against VgrG1 and Hcp2, respectively. Loading control (LC), visualized as trihalo compounds' fluorescence of the immunoblot membrane, is shown for total protein lysates. Phenamil (20 µM) was added to induce surface sensing in suspension. The experiment was repeated three times with similar results. Results from a representative experiment are shown. **b,** Viability counts of *E. coli* prey before (0 h) and after (4 h) co-incubation with the indicated *V. parahaemolyticus* BB22OP attackers at 30ºC (n = 3 co-cultures). Δ*hcp1* was used as a T6SS1$^-$ control. Asterisks denote the statistical significance between samples at the 4 h timepoint by unpaired, two-tailed Student's *t*-test (** $P < 0.005$; *** $P < 0.0005$); n.s., no significant difference ($P > 0.05$). **c,** Growth of *V. parahaemolyticus* BB22OP deletion strains in MLB (LB with 3% NaCl) at 30ºC is shown as OD_{600} measurements. Data represent the mean ± S.D. (n=4). **d,**

Viability counts of *E. coli* prey before (0 h) and after (4 h) co-incubation with the indicated *V. parahaemolyticus* BB22OP attackers deleted for *hcp2* (deletion that inactivates T6SS2 and allows monitoring only T6SS1-mediated antibacterial toxicity) at 30ºC (n = 3 co-cultures). Δ*hcp1* was used as a $T6SS1^-$ control. Asterisks denote the statistical significance between samples at the 4 h timepoint by unpaired, two-tailed Student's *t*-test (*** $P < 0.00005$); n.s., no significant difference ($P > 0.05$). Source data are provided as a source data file.

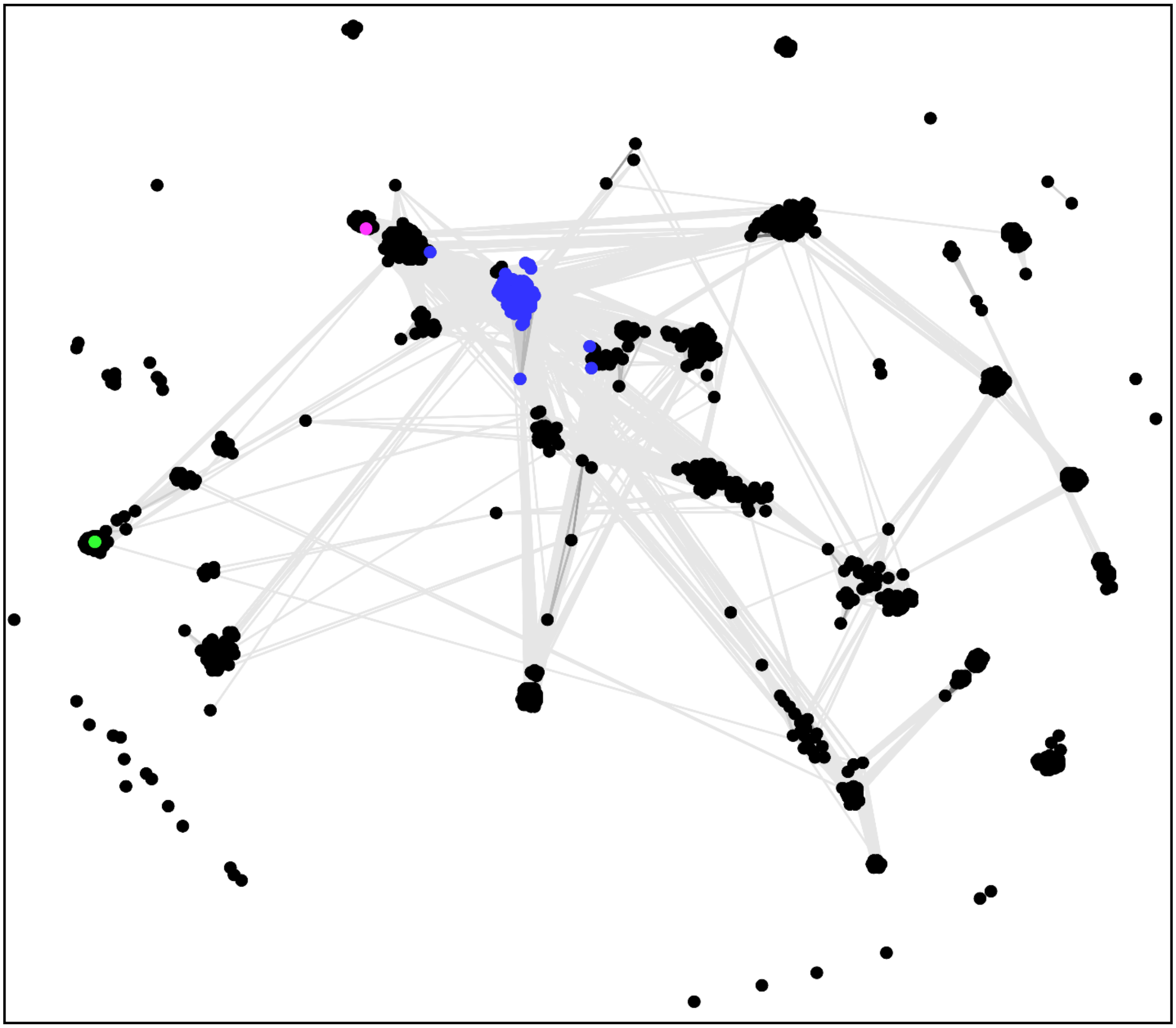

Supplementary Figure 5. Tme immunity genes encode a diverse family of mainly DUF1240-associated proteins. Proteins encoded immediately downstream of the Tme-encoding genes (i.e., Tmi) were clustered based on all-against-all sequence similarity (using CLANS). Circles denote individual proteins and connecting lines denote sequence similarity. Blue circles denote proteins containing a DUF1240 domain. Tmi1 and Tmi2 are denoted in green and magenta circles, respectively.

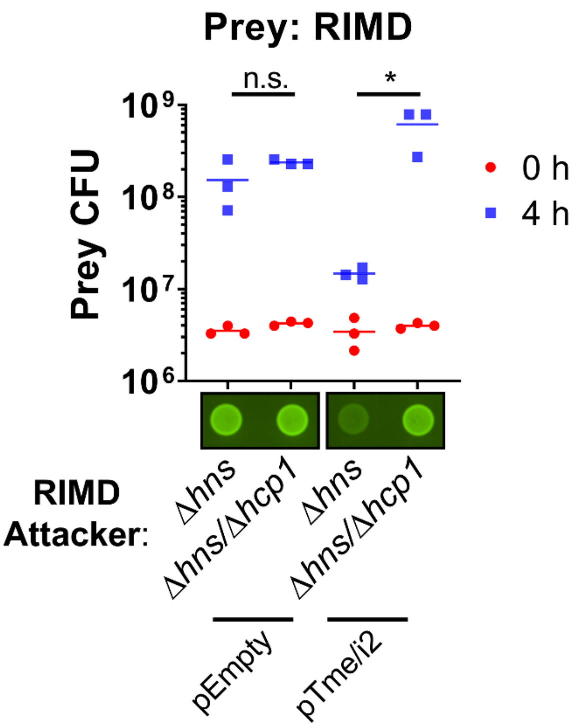

Supplementary Figure 6. *V. parahaemolyticus* T9109 Tme/i2 function as a T6SS1 E/I pair in the surrogate system. Viability counts of *V. parahaemolyticus* RIMD 2210633 parental prey (RIMD) before (0 h) and after (4 h) co-incubation with surrogate system *V. parahaemolyticus* RIMD 2210633 attacker strains, Δ*hns* (T6SS1^{+}) and Δ*hns*/Δ*hcp1* (T6SS1^{-}), on media containing L-arabinose at 30ºC (n = 3 co-cultures). Attackers harbor plasmids for the arabinose-inducible expression of Tme/i2 (pTme/i2) or an empty vector (pEmpty). Asterisk denotes statistical significance between samples at the 4 h timepoint by unpaired, two-tailed Student's *t*-test (P = 0.025); n.s., no significant difference $P > 0.05$). Images of representative spots of bacterial co-cultures, in which the indicated attacker strains were mixed with a parental RIMD 2210633 strain constitutively expressing GFP, are shown below. Survival of GFP-expressing prey was qualitatively assessed by monitoring GFP fluorescence. Source data are provided as a source data file.

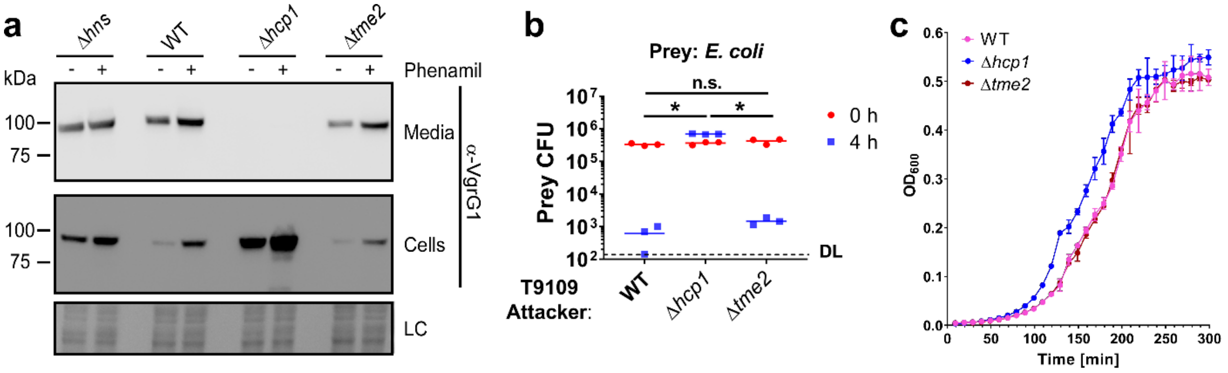

Supplementary Figure 7. T9109 T6SS1 is an antibacterial system that functions under warm marine-like conditions. a, Expression (cells) and secretion (media) of VgrG1 from the indicated *V. parahaemolyticus* T9109 mutants were detected by immunoblotting using specific antibodies against VgrG1. Loading control (LC), visualized as trihalo compounds' fluorescence of the immunoblot membrane, is shown for total protein lysates. Phenamil (20 µM) was added to induce surface sensing in suspension. **b,** Viability counts of *E. coli* prey before (0 h) and after (4 h) co-incubation with the indicated *V. parahaemolyticus* T9019 attackers at 30ºC. Δ*hcp1* was used as a T6SS1^{-} control. DL, the detection limit, is denoted as a dashed line. Asterisks denote statistical significance between samples at the 4 h timepoint by an unpaired, two-tailed Student's *t*-test (P = 0.0000007); n.s., no significant difference (P > 0.05). **c,** Growth of *V. parahaemolyticus* T9109 deletion strains in MLB (LB with 3% NaCl) at 30ºC is shown as OD_{600} measurements. Data represent the mean ± S.D. (n=3). Source data are provided as a source data file.

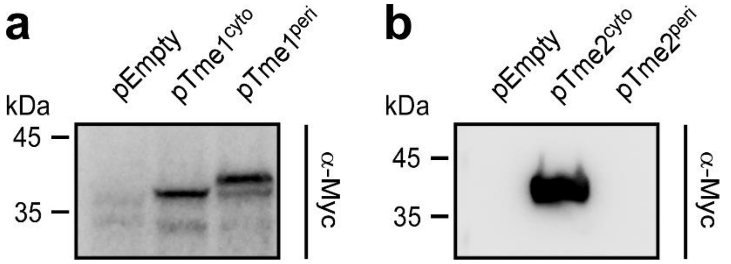

Supplementary Figure 8. The expression of Tme1 and Tme2 in *E. coli*. The expression of Myc-tagged cytoplasmic (cyto) or periplasmic (peri) forms of Tme1 **(a)** and Tme2 **(b)** in *E. coli* BL21 (DE3) was detected by immunoblotting using anti-Myc antibodies. Loading control (LC), visualized as trihalo compounds' fluorescence of the immunoblot membrane, is shown for total protein lysates. Source data are provided as a source data file. The experiments were repeated three times with similar results. Results from a representative experiment are shown.

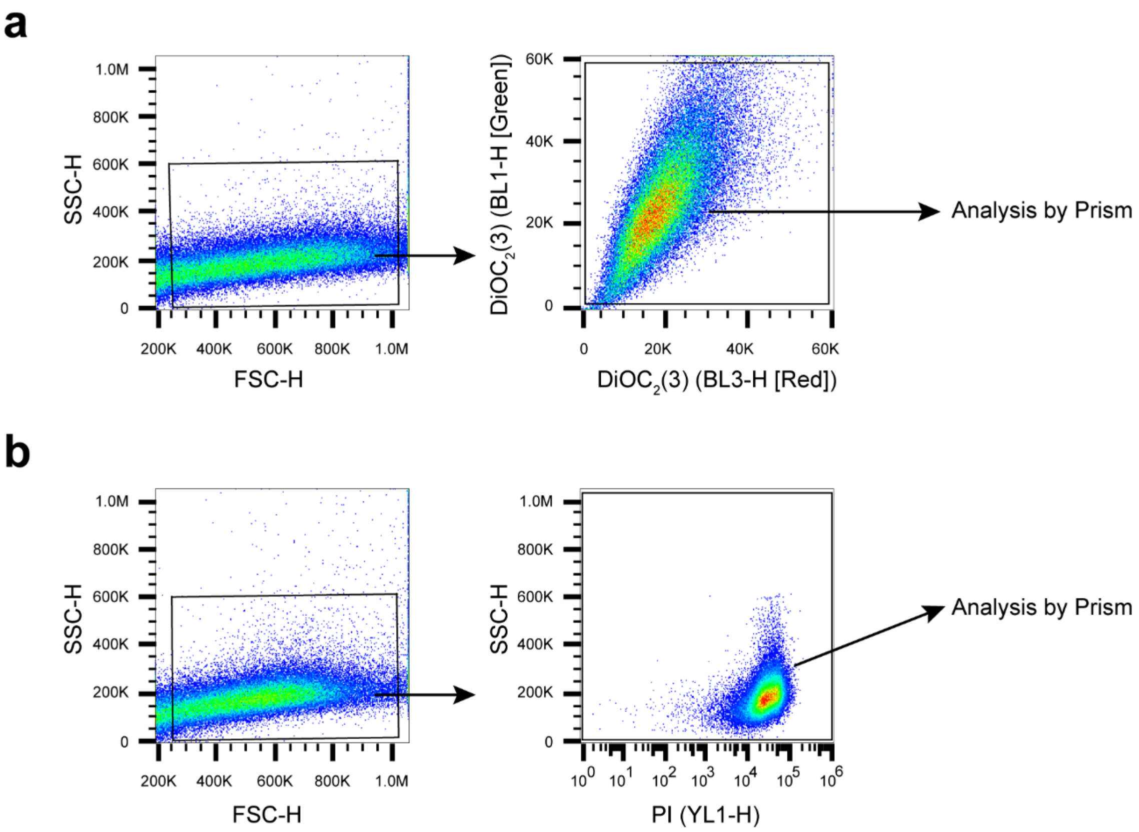

Supplementary Figure 9. Flow cytometry gating strategy. Data in Figure 6e and 6f were analysed using flow cytometry as shown. A minimum of 50,000 bacteria were first gated using forward and side scatter. **a,** $DiOC_2(3)$ was excited using Blue laser (488 nm) and emission was detected using the 530/30 (BL1-H [green]) and 590/40 (BL3-H [red]) filters. Red/green ratios of gated bacteria were calculated from the geometric mean fluorescence intensity (MFI) of each channel using FlowJo V10 software. **b,** PI was excited using Yellow laser (561 nm) and emission was detected using the 585/15 (YL-1-H) filter. Geometric mean fluorescence intensity (MFI) was calculated using FlowJo V10 software.

Supplementary Table 1. List of oligonucleotide primers used in this study for plasmid construction.

Primer	Sequence [a, b]	Plasmid backbone	Constructed plasmid name
14465/0_F	gctaacaggaggaattaaccATGTTAGGTAATGATATTTTTGAAG	pBAD	pVp14465/0
VgrG1b_14465/0_F	gctaacaggaggaattaaccATGGTTAATGATGTAAACTTCAC	pBAD	p1b+Vp14465/0
14465/0_R	ttttgttcgggcccaagcttAGCTTCCTGTAAAACCCAAAAG	pBAD	pVp14465/0 and p1b+Vp14465/0
VPBB_RS00910-05_F	gctaacaggaggaattaaccATGAGCAATAAAACCAGC	pBAD	pVPBB_RS00910-15
VPBB_RS00910-05_R	ttttgttcgggcccaagcttGAAGGTGTAGACAAAGGTG		
VPBB_RS13115-20_F	gctaacaggaggaattaaccATGAGTGCTATCACCATC	pBAD	pVPBB_RS13115-20
VPBB_RS13115-20_R	ttttgttcgggcccaagcttTTCCACTGCCTCCACTGTTTTAGC		
VPBB_RS13120-25_F	gctaacaggaggaattaaccATGATCGACCTTGAAACC	pBAD	pVPBB_RS13120-25
VPBB_RS13120-25_R	ttttgttcgggcccaagcttTGCCGCCACCTCACTAATATTC		
VPBB_RS13145-50_F	gctaacaggaggaattaaccATGAGTTATTTAGGAGCAAAAGG	pBAD	pVPBB_RS13145-50
VPBB_RS13145-50_R	ttttgttcgggcccaagcttGAGTTCTTTTTCTCCTTTAG		
VPBB_RS13210-15_F	gctaacaggaggaattaaccATGCGCCGCTGGCCGAAC	pBAD	pVPBB_RS13210-15
VPBB_RS13210-15_R	ttttgttcgggcccaagcttCTCCCTTTGCTCAATCTC		
VPBB_RS13280-75_F	gctaacaggaggaattaaccATGAACAAAGAAGATCTAAAG	pBAD	pVPBB_RS13280-75
VPBB_RS13280-75_R	ttttgttcgggcccaagcttTTACCTACTTGAACGGTATATAT		
VPBB_RS15030-35_F	gctaacaggaggaattaaccATGAGATATGAATCCGCTC	pBAD	pVPBB_RS15030-35 and pTme1^{Cyto}
VPBB_RS15030-35_R	ttttgttcgggcccaagcttTCGAGCTTTAACGTACATCAAG	pBAD	pVPBB_RS15030-35
Tme1_Cyto_R	ttttgttcgggcccaagcttTATCGCACTTTCTAACCTTTC	pBAD	pTme1^{Cyto}
VPBB_RS18960-65	gctaacaggaggaattaaccATGGGAAAAATTGTATTTG	pBAD	pVPBB_RS18960-65
VPBB_RS18960-65_R	ttttgttcgggcccaagcttCTTCAATTTGCCGTTTATAAAG		
Tme/i2_F	gctaacaggaggaattaaccATGAGTAACCCAGAAAGC	pBAD	pTme/i2 and pTme2 (pTme2^{Cyto})
Tme/i2_R	ttttgttcgggcccaagcttCTAGTGATTCCTTATTCG	pBAD	pTme/i2
Tme2_R	ttttgttcgggcccaagcttTAATATTTTTGGTGTCAATTCATTTAACG	pBAD	pTme2 (pTme2^{Cyto})
Tme1_Cyto_R	ttttgttcgggcccaagcttTATCGCACTTTCTAACCTTTC	pBAD	pTme1^{Cyto}
Tme1_Peri_F	cccagccggcgatggccATGAGATATGAATCCGCTCCTGTAGCCCC	pPER5	pTme1^{Peri}

Tme1_Peri_R	ttttgttcgggcccaagcttTATCGCACTTTCTAACCTTTCAACTATCGTTTGAG		
Tme2_Peri_F	cccagccggcgatggccATGAGTAACCCAGAAAGCATATTTCCTAAAGGCTC	pPER5	pTme2^{Peri}
Tme2_Peri_R	ttttgttcgggcccaagcttTAATATTTTTGGTGTCAATTCATTTAACGATTTTTTGATGAATCG		
VasX_Peri_F	cccagccggcgatggccATGAGTAATCCCAATCAAG	pPER5	pVasX^{Peri}
VasX_Peri_R	ttttgttcgggcccaagcttACCTTTTCCTACAACGAGATTTCTTGTG		
Tme1_pBAD33.1_F	ctttaagaaggagatatacatATGAGATATGAATCCGCTCCTGTAGCCCC	pBAD33.1	pTme1
Tme1_pBAD33.1_R	cgtcgtcatccttgtaatcTATCGCACTTTCTAACCTTTCAACTATCGTTTGAG		
Tmi1_F	ctttaagaaggagatatacatGTGCGATATAAAACTAAAATCCG	pBAD33.1	pTmi1
Tmi1_R	cgtcgtcatccttgtaatcTCGAGCTTTAACGTACATC		
Tmi2_F	ctttaagaaggagatatacatATGAGCGACTCCCAATTG	pBAD33.1	pTmi2
Tmi2_R	cgtcgtcatccttgtaatcGTGATTCCTTATTCGACATAAAG		
Hcp1_SacI_UP_F	caaagagctcCTGTCGTGAACTTGCTCAG	pDM4	pDM4: *hcp1* (from BB22OP and T9109)
Hcp1_BamHI_UP_R	caacggatccCGCTATTTCCTTTTCTAAAATCTG		
Hcp1_BamHI_DN_F	caccggatccTTGCTTTTTGCGTAAAGATTCAGG		
Hcp1_SalI_DN_R	caaagtcgacCTGTAACCAGACGCCAAACG		
Hns_SacI_UP_F	cacagagctcTACCTACACCAAGAGAAAC	pDM4	pDM4: *hns* (from BB22OP and T9109)
Hns_UP_R	TTTAAACAAGAACGAAACGATTCCTATTAATAGGTTAACG		
Hns_DN_F	TTAATAGGAATCGTTTCGTTCTTGTTTAAAAAGGCTCCCG		
Hns_SalI_DN_R	caaagtcgacGTGTTGATGGTGCTATCG		
Hcp2_SalI_UP_F	caaagtcgacTATGGGAGCGAAGACAAC	pDM4	pDM4: *hcp2* (BB22OP)
Hcp2_BamHI_UP_R	caaaggatccGCTAATCTCCTAGAGCATTATTAATTGAC		
Hcp2_BamHI_DN_F	cagaggatccTTTATTGTGCGGAGGGGTTATC		
Hcp2_XbaI_DN_R	cagatctagaTCCGCTACATAGATAAACGTTCTG		
Tme1_SacI_UP_F	cacagagctcCTTTGAATGTTGTGAGTG	pDM4	pDM4: *tme1* and pDM4: *tme/i1*
Tme1_BamHI_UP_R	caacggatccGGAAGTCGGTTTTACTGATAAATAAAAACG		
Tme1_BamHI_DN_F	cactggatccCGATAGTTGAAAGGTTAG	pDM4	pDM4: *tme1*
Tme1_SalI_DN_R	caatgtcgacTTTTCTGCCGTCTCTCAG		

Tmi1_BamHI_DN_F	caccggatccTTGAACTTAACGGCACTTAATC	pDM4	pDM4: *tme/i1*
Tmi1_SalI_DN_R	caatgtcgacGCTGTAATACCTTAGCAAC		
Tme2_SacI_UP_F	catagagctcTCACTCTTGAACAGTTTG	pDM4	pDM4: *tme2* and pDM4: *tme/i2*
Tme2_BamHI_UP_R	cacaggatccGGTTATTGCTACGAAATG		
Tme2_BamHI_DN_F	caccggatccGCGACTCCCAATTGATAAAC	pDM4	pDM4: *tme2*
Tme2_SalI_DN_R	cacagtcgacACCTCAAATACAGAAACGGG		
Tmi2_BamHI_DN_F	caccggatccCTATATCGAACCAGTTAG	pDM4	pDM4: *tme/i2*
Tmi2_SalI_DN_R	cacagtcgacTTTTGAGTCTTAGCTCAC		

[a] Uppercase letters correspond to gene sequences; lowercase letters correspond to plasmid sequences.

[b] Restriction enzyme recognition sites are underlined
