# Supplementary material for: A comparative genomics methodology reveals a widespread family of membrane-disrupting T6SS effectors
Source: Nat Commun. 2020 Feb 27;11:1085. doi: 10.1038/s41467-020-14951-4 (PMC7046647; doi:10.1038/s41467-020-14951-4)
Supplement: Supplementary file 3 — Description of Additional Supplementary Files [file 41467_2020_14951_MOESM3_ESM.pdf]

Description of Additional Supplementary Files

File Name: Supplementary Data 1
Description: OrthoANI analysis of *V. parahaemolyticus* genomes.

File Name: Supplementary Data 2
Description: Summary of T6SS core proteins identified in *V. parahaemolyticus* genomes.

File Name: Supplementary Data 3
Description: Comparative genomics analysis of *V. parahaemolyticus* BB22OP proteins.

File Name: Supplementary Data 4
Description: *V. parahaemolyticus* BB22OP effector candidates identified by comparative genomic analysis.

File Name: Supplementary Data 5
Description: Tme-containing proteins and adjacently encoded proteins.

File Name: Supplementary Data 6
Description: Summary of T6SS core proteins identified in genomes harboring Tme-containing proteins.
