# Supplementary material for: A comparative genomics methodology reveals a widespread family of membrane-disrupting T6SS effectors
Source: Nat Commun. 2020 Feb 27;11:1085. doi: 10.1038/s41467-020-14951-4 (PMC7046647; doi:10.1038/s41467-020-14951-4)
Supplement: Supplementary file 10 — Reporting Summary [file 41467_2020_14951_MOESM10_ESM.pdf]

nature research

Corresponding author(s): Dor Salomon and Eran Bosis

Last updated by author(s): Feb 4, 2020

Reporting Summary

Nature Research wishes to improve the reproducibility of the work that we publish. This form provides structure for consistency and transparency in reporting. For further information on Nature Research policies, see Authors & Referees and the Editorial Policy Checklist .

Statistics

For all statistical analyses, confirm that the following items are present in the figure legend, table legend, main text, or Methods section.

n/a	Confirmed	
☐	☒	The exact sample size (*n*) for each experimental group/condition, given as a discrete number and unit of measurement
☐	☒	A statement on whether measurements were taken from distinct samples or whether the same sample was measured repeatedly
☐	☒	The statistical test(s) used AND whether they are one- or two-sided<br>*Only common tests should be described solely by name; describe more complex techniques in the Methods section.*
☒	☐	A description of all covariates tested
☐	☒	A description of any assumptions or corrections, such as tests of normality and adjustment for multiple comparisons
☐	☒	A full description of the statistical parameters including central tendency (e.g. means) or other basic estimates (e.g. regression coefficient) AND variation (e.g. standard deviation) or associated estimates of uncertainty (e.g. confidence intervals)
☐	☒	For null hypothesis testing, the test statistic (e.g. *F*, *t*, *r*) with confidence intervals, effect sizes, degrees of freedom and *P* value noted<br>*Give P values as exact values whenever suitable.*
☒	☐	For Bayesian analysis, information on the choice of priors and Markov chain Monte Carlo settings
☒	☐	For hierarchical and complex designs, identification of the appropriate level for tests and full reporting of outcomes
☒	☐	Estimates of effect sizes (e.g. Cohen's *d*, Pearson's *r*), indicating how they were calculated

Our web collection on statistics for biologists contains articles on many of the points above.

Software and code

Policy information about availability of computer code

Data collection	NCBI Blast+ 2.7.1 suite
Data analysis	CD-Hit v4.7, Phobius v1.01, SignalP 5.0, HHpred, Jpred 4.0, WebLogo 3.0, MEGA 7 (including MUSCLE), CLANS, GraphPad Prism 8, FlowJo V10, OrthoANI algorithm

For manuscripts utilizing custom algorithms or software that are central to the research but not yet described in published literature, software must be made available to editors/reviewers. We strongly encourage code deposition in a community repository (e.g. GitHub). See the Nature Research guidelines for submitting code & software for further information.

Data

Policy information about availability of data

All manuscripts must include a data availability statement. This statement should provide the following information, where applicable:

- Accession codes, unique identifiers, or web links for publicly available datasets
- A list of figures that have associated raw data
- A description of any restrictions on data availability

The experimental and computational data that support the findings of this research are available in this article and its supplementary information files, or upon request from the corresponding authors. The source data underlying Figs. 2, 3b-d, 5b-d, 6a-f, and Supplementary Figs. 2a-c, 3, 4a-d, 6, 7a-c, 8a-b, are provided as a Source Data file.

Field-specific reporting

Please select the one below that is the best fit for your research. If you are not sure, read the appropriate sections before making your selection.

Life sciences study design

All studies must disclose on these points even when the disclosure is negative.

Sample size	No sample size calculations were performed. All experiments are performed on bacterial cultures.
Data exclusions	No data were excluded
Replication	Experiments were repeated at least 3 times with similar results, as noted in the Methods section. Attempts at replication were successful.
Randomization	No randomization was performed
Blinding	No blinding was performed

Reporting for specific materials, systems and methods

We require information from authors about some types of materials, experimental systems and methods used in many studies. Here, indicate whether each material, system or method listed is relevant to your study. If you are not sure if a list item applies to your research, read the appropriate section before selecting a response.

Materials & experimental systems

n/a	Involved in the study
☐	☒ Antibodies
☒	☐ Eukaryotic cell lines
☒	☐ Palaeontology
☒	☐ Animals and other organisms
☒	☐ Human research participants
☒	☐ Clinical data

Methods

n/a	Involved in the study
☒	☐ ChIP-seq
☐	☒ Flow cytometry
☒	☐ MRI-based neuroimaging

Antibodies

Antibodies used	1) c-Myc Antibody (9E10): sc-40; Santa Cruz Biotechnology; Lot#: F1917.<br>2) DYKDDDDK Tag (D6W5B) Rabbit mAb #14793; Lot#: 4<br>3) anti-VgrG1; Polyclonal antibodies were produced in-house with rabbits for the Vibrio parahaemolyticus VgrG1 (VP1394) peptide KDMSTKVLNNRYRDIGQDE and were affinity purified (Thermo Fisher)<br>4) anti-Hcp2; Polyclonal antibodies were produced in-house with rabbits for the Vibrio parahaemolyticus Hcp2 (VPA1027) peptide KYADIKGEATAEQ and were affinity purified (Thermo Fisher)
Validation	1) c-Myc Antibody manufacturer website: https://www.scbt.com/scbt/product/c-myc-antibody-9e10<br>2) DYKDDDDK Tag (D6W5B) Antibody manufacturer website: Cell Signaling Technology; https://www.cellsignal.com/products/primary-antibodies/dykddddk-tag-d6w5b-rabbit-mab-binds-to-same-epitope-as-sigma-s-anti-flag-m2-antibody/14793<br>3) VgrG1 Antibody specificity was tested in-house using immunoblots withVibrio wild-type or vgrG1-deletion strains. This antibody was also used in DOI: 10.1128/AEM.00737-17, DOI: 10.15252/embr.201744226, and in DOI: 10.1038/s41467-019-11546-6<br>4) Hcp2 Antibody specificity was tested in-house using immunoblots with Vibrio wild-type or hcp2-deletion strains. This antibody was also used in DOI: 10.1128/AEM.00737-17

Flow Cytometry

Plots

Confirm that:

☒ The axis labels state the marker and fluorochrome used (e.g. CD4-FITC).

☒ The axis scales are clearly visible. Include numbers along axes only for bottom left plot of group (a 'group' is an analysis of identical markers).

☒ All plots are contour plots with outliers or pseudocolor plots.

☒ A numerical value for number of cells or percentage (with statistics) is provided.

Methodology

Sample preparation	Detailed in Methods section
Instrument	ThermoFisher Scientific Attune NxT flow cytometry
Software	FlowJo V10
Cell population abundance	Minimum of 50,000 bacteria were gated in each sample
Gating strategy	Detailed in Methods section and in Supplementary Figure 9

☒ Tick this box to confirm that a figure exemplifying the gating strategy is provided in the Supplementary Information.
